# A Disintegrin and Metalloproteases (ADAMs): Activation, Regulation and Mechanisms of Catalysis

**DOI:** 10.3390/ijms22168762

**Published:** 2021-08-16

**Authors:** Thorsten Maretzky

**Affiliations:** Inflammation Program and Department of Internal Medicine, Roy J. and Lucille A. Carver College of Medicine, University of Iowa, Iowa City, IA 52242, USA; thorsten-maretzky@uiowa.edu

In the late 1980s, Paul Primakoff and colleagues showed that fertilization could be blocked in an in vitro sperm–egg fusion assay by inoculating them in the presence of a disintegrin and metalloprotease (ADAM)-specific antibody. This elegant experiment revealed the adhesive properties of these single-transmembrane spanning proteins and laid the foundation for several groundbreaking discoveries in the field of ADAM biology in the following years. While several ADAM proteins presumably function as adhesion molecules, some members of this family are catalytically active and regulate diverse biological processes, including neuronal differentiation, muscular tissue formation, inflammation and immune responses [1]. Nearly 40 years later, the complexity of their substrate portfolio and overall proteolytic capacities are much better understood. We have also begun to appreciate that the regulatory mechanisms of their catalytic activities are multifarious and influenced not only by diverse signaling pathways but also depend on the precise interplay between the protease, its substrates and specific support from assisting proteins.

In this Special Issue, we survey the recent advances in this field, adding formerly unknown insight into the aspects of ADAM biology, activity and substrate specificity. We have collected a series of research reports and review articles from prominent authors in the field, which explore the functional role of a variety of catalytically active ADAM proteases and their substrates, ranging from ADAMs 9, 10, 12, 15 and 17 to components of their substrate network.

As it became more and more evident in the early 2000s that ADAM proteases serve as important molecular switches in various signaling circuits on the cell surface and in the extracellular space, the search for their substrates was rapidly expanding [2].

Within this framework, we aimed to decipher the nature of the proteases that are responsible for the proteolytic release (ectodomain shedding) of a family of fibroblast growth factor receptors (FGFRs) from the cell surface. These receptor tyrosine kinases have been implicated in various cellular processes, such as embryonic development and wound healing, but have also been associated with enhanced tumor survival, angiogenesis and metastases [3]. Interestingly, we found that the ectodomain shedding of FGFRs is mediated by two distinct members of the disintegrin and metalloprotease family, ADAM10 and ADAM17 [4].

Besides identifying the proteases involved in the release of these cell surface receptors, the functional consequences of this ADAM-mediated ectodomain shedding should be evaluated as well. In this regard, Anne Hanneken and colleagues examined the biological properties of FGFR1 ectodomain shedding and how this cleavage event is regulated. The authors not only found that FGFR1 is constitutively cleaved but that this released soluble form also inhibits FGF2-induced proliferation and competes with membrane-bound FGFR for ligand binding. Furthermore, the authors showed that the underlying shedding mechanism of FGFR1 shedding is regulated by two distinct signaling pathways [5]. In summary, these studies suggest that shed forms of FGFRs are biologically active and are potentially functioning as natural inhibitors of FGF-mediated signaling events.

While we continue to discover novel substrates for these proteases, other ADAM substrates have been characterized in greater detail in recent years. Some of the presumably most extensively studied and best characterized ADAM substrates are the Notch receptors [6]. Notch signaling is critical for cellular differentiation and cell-fate determination during development and homeostasis. Several studies have shown that ADAM10 cleaves the Notch receptor Notch1 under physiological, ligand-dependent conditions, while ADAM17 mostly cleaves Notch1 under ligand-independent conditions. However, the underlying mechanisms that control the distinct contributions of these ADAMs in Notch processing remain less well understood. By using an elegant in vitro model system, Rolake Alabi and colleagues investigated the relative contributions of ADAMs 10 and 17 in ligand-dependent or ligand-independent Notch processing. Specifically, they showed that EDTA-stimulated ADAM17-dependent Notch1 processing is rapid, whereas the Delta-like 4-induced ligand-dependent Notch1 cleavage is slower and depends on ADAM10 [7].

ADAM10 has also captured scientific interest because of its pivotal role in the non-amyloidogenic processing of the amyloid-β precursor protein in neurons, inhibiting the production of β-amyloid peptide in Alzheimer’s disease (AD). An interesting study, conducted by Izabela Vatanabe and colleagues, determined the activity of this protease in the plasma and cerebrospinal fluids of patients with amnestic mild cognitive impairment and mild AD [8]. Considering the importance of the localization of ADAM10 for its catalytic function, the authors found that plasma levels of ADAM10 are significantly increased in patients with mild AD. In addition, they found that these soluble forms of ADAM10 are inactive, suggesting that the protease requires its transmembrane domain to be catalytically active. Remarkably, these results suggest the potential use of plasma ADAM10 as a predictive biomarker, even though further studies are warranted prior to clinical use.

Many other chronic conditions, such as rheumatoid arthritis and systemic lupus erythematosus (SLE), as well as various types of cancers, have also been linked to aberrant ADAM expression and activity [1]. Often, this has been correlated with increased levels of shed, soluble forms of growth factor ligands and inflammatory markers. Particularly in SLE, elevated levels of soluble CD137 have been involved in both the development and progression of the disease. Jana Seidel and colleagues present evidence that the membrane-bound form of CD137 is shed from primary T cells by ADAM10 and possibly ADAM17 as well [9]. Furthermore, soluble CD137 is functionally active and promotes T cell proliferation.

Of note, a thorough analysis of additional substrates cleaved by ADAMs 10 and 17 and their activities in subcellular sites and multiprotein complexes is presented in the review article by Francesca Tosetti and colleagues [10].

In addition, several studies have linked elevated levels of ADAM17 and connective tissue growth factor (CTGF) with the development of idiopathic pulmonary fibrosis. However, the underlying signaling cascades responsible for this increased expression in this malignancy remain poorly defined. Shu-Ching Ou and colleagues point to novel evidence regarding the impact of transforming growth factor-β (TGF-β)-mediated signaling pathways on the induced expression of CTGF in lung epithelial cells, uncovering the importance of ADAM17 as well as of ERK, RSK1 and C/EBPβ, which are well known for their functional role in epithelial–mesenchymal transition (EMT) [11]. Interestingly, TGF-β also enhances fibronectin expression, an EMT marker, and can be reduced by inhibiting ADAM17 function. 

Although ADAMs have been consistently associated with the development of several chronic diseases, they can also contribute to the detrimental effects in acute pathologies. In this context, ADAM17 has been shown to play a critical role in the pathogenesis of sepsis, an acute condition caused by a dysregulated response to infection. With a mortality rate of nearly 50%, there is an urgent need for the identification of novel therapeutic avenues for severe sepsis treatment. Towards this direction, Hemant Mishra and colleagues examined the effects of an ADAM17 function blocking monoclonal antibody in the pathogenesis of sepsis [12]. In an in vivo model of polymicrobial sepsis, mice treated with this biologics-based approach show a significantly increased rate of recovery. Remarkably, its beneficial potential can be further improved when the antibody treatment is combined with antibiotic administration.

Besides modulating disease-relevant processes, ADAM proteins also regulate important physiological functions such as cell–cell interactions and the activation of various signaling pathways, as well as cell proliferation and differentiation [13]. Anke Seifert and colleagues investigated the role of ADAM17 and its regulatory binding partner iRhom2 in the context of bacterial uptake by mononuclear phagocytes [14]. Interestingly, deletion of either iRhom2 or ADAM17 in these cells leads to increased internalization of Gram-negative and -positive bacteria. This enhanced bacterial phagocytosis depends on TNF production and is accompanied by increased levels of the chemokine CXCL8, but does not appear to affect cell surface expression of other interaction partners of phagocytosis.

In addition, previous studies have shown an essential role for ADAM17 in differentiation of hypertrophic chondrocytes during endochondral ossification. In this process, growing cartilage is gradually replaced by bone to form the skeletal system. Renpeng Fang and colleagues point to novel evidence regarding the impact of iRhoms 1 and 2 in endochondral ossification, uncovering the importance of these inactive rhomboids in the regulation of bone growth by controlling the maturation of ADAM17 during differentiation of hypertrophic chondrocytes [15]. Particularly, iRhom2-deficient mice with a targeted deletion of iRhom1 in chondrocytes show retarded bone growth caused by a significantly expanded zone of hypertrophic mineralizing chondrocytes in the growth plate. The observed enlarged zone of mineralized hypertrophic chondrocytes in these mice closely resembles the abnormal growth plate in transforming growth factor α (TGFα)-deficient mice. These findings are quite fascinating and support a model in which iRhoms 1 and 2 direct bone growth by controlling ADAM17-dependent TGFα shedding and the subsequent EGFR signaling axis in chondrocytes and endochondral ossification.

An excellent analysis of the signaling processes controlled by iRhoms and their regulatory role in immune responses to infections is presented in the review article by Mazin Al-Salihi and Philipp Lang [16]. In addition, the authors highlight that a thorough understanding of how iRhoms modulate ADAM17 activity in human diseases is an essential requirement regarding the validation of these intramembrane proteins as promising therapeutic targets for autoimmune diseases and cancer therapy.

While iRhoms 1 and 2 specifically interact with ADAM17, the tissue inhibitor of metalloprotease 3 (TIMP3) forms complexes with either matrix metalloproteases (MMPs) or ADAM proteins. Decreased levels of TIMP3 have been associated with aneurysms, cardiac inflammation and atherosclerosis, suggesting the potential utility of targeted TIMP3 delivery to inhibit aberrant metalloprotease activity in these diseases. Towards this direction, Anna Carreca and colleagues developed a high-resolution mass spectrometry-based approach to evaluate changes induced by stable overexpression of TIMP3 in the cell surface proteome [17]. The profiling of cell membrane proteins revealed TIMP3-induced changes in the protein composition of the cell surface of TIMP3-overexpressing cells as well as potentially novel ADAM substrates, including ADAM15.

Similarly to iRhoms 1 and 2, a subgroup of tetraspanins, TspanC8, function as ADAM10 interaction partners and support its regulation and maturation. The role of this tetraspanin family in substrate recognition and their therapeutic potential are further discussed in a comprehensive and informative review article by Neale Harrison and Michael Tomlinson in this Special Issue [18]. 

Though several studies herein have focused on the biology of ADAMs 10 and 17 specifically, little attention has been given to the variety of physiological functions and pathophysiological conditions that are influenced by other catalytically active ADAM proteins. An interesting article, written by Cheng-Wei Chou and colleagues, effectively summarizes the existing literature regarding the role of ADAM9 in developmental processes as well as in inflammation and degenerative diseases [19]. Moreover, recent advances in therapeutic strategies targeting ADAM9-related pathways are discussed as well.

In conclusion, the scientific discoveries presented in this Special Issue should inform future research and intervention initiatives and draw closer attention to the clinical importance of these ubiquitous molecular scissors, laying the foundation for treatment strategies to target ADAM-dependent signaling pathways that cause sustained inflammation and an increased risk for cancer as well as therapies to treat chronic diseases such as Alzheimer’s, cardiovascular disorders and cancer.
